# Clinical value of plasma and peripheral blood mononuclear cells Epstein–Barr Virus DNA dynamics on prognosis of allogeneic stem cell transplantation

**DOI:** 10.3389/fcimb.2022.980113

**Published:** 2022-09-16

**Authors:** Xi Zhou, Xuan Lu, Jing He, Ziwei Xu, Qian Li, Pian Ye, Zhaodong Zhong, Wei Shi, Han Yan, Yong You, Yu Hu, Huafang Wang

**Affiliations:** ^1^ Institute of Hematology, Union Hospital, Tongji Medical College, Huazhong University of Science and Technology, Wuhan, China; ^2^ Department of Infectious Diseases, Union Hospital, Tongji Medical College, Huazhong University of Science and Technology, Wuhan, China

**Keywords:** allo-HSCT, Epstein-Barr virus DNA, peripheral blood mononuclear cells, plasma, posttransplant lymphoproliferative disorders, immune reconstitution, prognosis

## Abstract

The application of intracellular and extracellular Epstein–Barr virus (EBV) DNA in allogeneic hematopoietic stem cell transplantation (allo-HSCT) has been poorly characterized. We conducted a combined prospective-retrospective study of 300 patients who underwent allo-HSCT between 2016 to 2019 in our center and monitored for EBV DNA within the first year after HSCT. Combining the optimal cut-off value of EBV DNA load (7.3×10^4^ copies/10^6^ cells) in peripheral blood mononuclear cells (PBMCs) and qualitative detection in plasma (400 copies/mL) allowed for the better differentiation of EBV-related posttransplant lymphoproliferative disorders (EBV-PTLD), with increased sensitivity (100%) and specificity (86%), and provided the effective risk stratification of EBV DNA level according to their impact on transplant outcomes. By multivariate analysis, patients with intermediate-level of EBV DNA load (low EBV DNA load in PBMCs or high load in PBMCs but negative in plasma) was associated with superior overall survival (HR 1.92, 95% CI 1.03-3.57, *p*=0.039) and lower transplant-related mortality (HR 3.35, 95% CI 1.31-8.58, *p*=0.012) compared to those with high-level (high load in PBMCs and positive in plasma). Notably, high EBV-level group had poor reconstitution of CD4+ and CD8+T cells, and both low and high EBV-level groups showed abnormally increase in IL-10 level within one year. Additionally, patients with peak EBV DNA load in PBMCs during 3-12 months had a higher incidence of chronic graft versus host disease (GVHD) than those within 3 months post transplantation (17.4% vs 13.7%, *p*=0.029). Collectively, EBV DNA in PBMCs can synergistically predict the risk of EBV-PTLD and GVHD. The intermediate-level of EBV DNA presented in plasma and PBMCs might contribute to a better reconstitution of T cells associated with favorable prognosis of allo-HSCT.

## Introduction

Allo-hematopoietic stem cell transplantation (allo-HSCT) is an effective therapeutic approach for many hematologic malignancies as well as immune diseases (Kołodziejczak et al., 2021). With the continuous improvement of conditioning regimens including particularly selective T‐cell depletion of the graft or selective pharmacologic induction of T‐cell dysfunction, especially in haploidentical HSCT, the occurrence and severity of graft versus host disease (GVHD) is effectively controlled (Yang et al., 2019). Nonetheless, Epstein–Barr Virus (EBV) reactivation due to immunosuppression is still a major concern affecting the outcome of allo-HSCT (Bender Ignacio et al., 2019), EBV drives the proliferation of infected lymphocytes in the setting of impaired cellular immunity, leading to the development of EBV‐PTLD (Nowakowska et al., 2015; Marcelis and Tousseyn, 2019), which mostly occurred in the first year following HSCT with a high mortality of 50%-90% (Rasche et al., 2014).

The non-invasive measurement for EBV DNA by quantitative real-time PCR allows the rapid monitoring of EBV reactivation and progression of this viral infection (Al Hamed et al., 2020). Growing data indicate the detection of cell‐free EBV DNA in plasma appears more specific for screening PTLD onset and other EBV-associated diseases and more commonly used as a clinical indicator of antiviral therapy decision making (Kanakry et al., 2016). However, given the relatively low sensitivity of plasma EBV DNA detection (Wareham et al., 2018; Kawada et al., 2019), and lymphocytes are the target cell for EBV infection, the quantitative EBV DNA determination in PBMCs among HSCT should not be overlooked (Ito et al., 2016; Münz, 2021). Nevertheless, the optimal thresholds as well as the point of time for post-transplant EBV DNA monitoring varies by different institutions, no generally accepted guideline for monitoring EBV DNA in HSCT has been established (Ruf et al., 2012; Chiereghin et al., 2019), studies to figure out these issues are warranted. Otherwise, the timing of preemptive treatment of PTLD is controversial (Walti et al., 2021), risk stratification based on EBV DNA load may contribute to clinical decision making for preemptive treatment. The primary goals of the study were to investigate optimal thresholds of EBV DNA especially in PBMCs which allowed better evaluation of EBV-PTLD onset and explore the effect of post-transplant EBV DNA level on immune status and prognosis of HSCT patients.

## Material and methods

### Study design and participants

**Figure 1:**
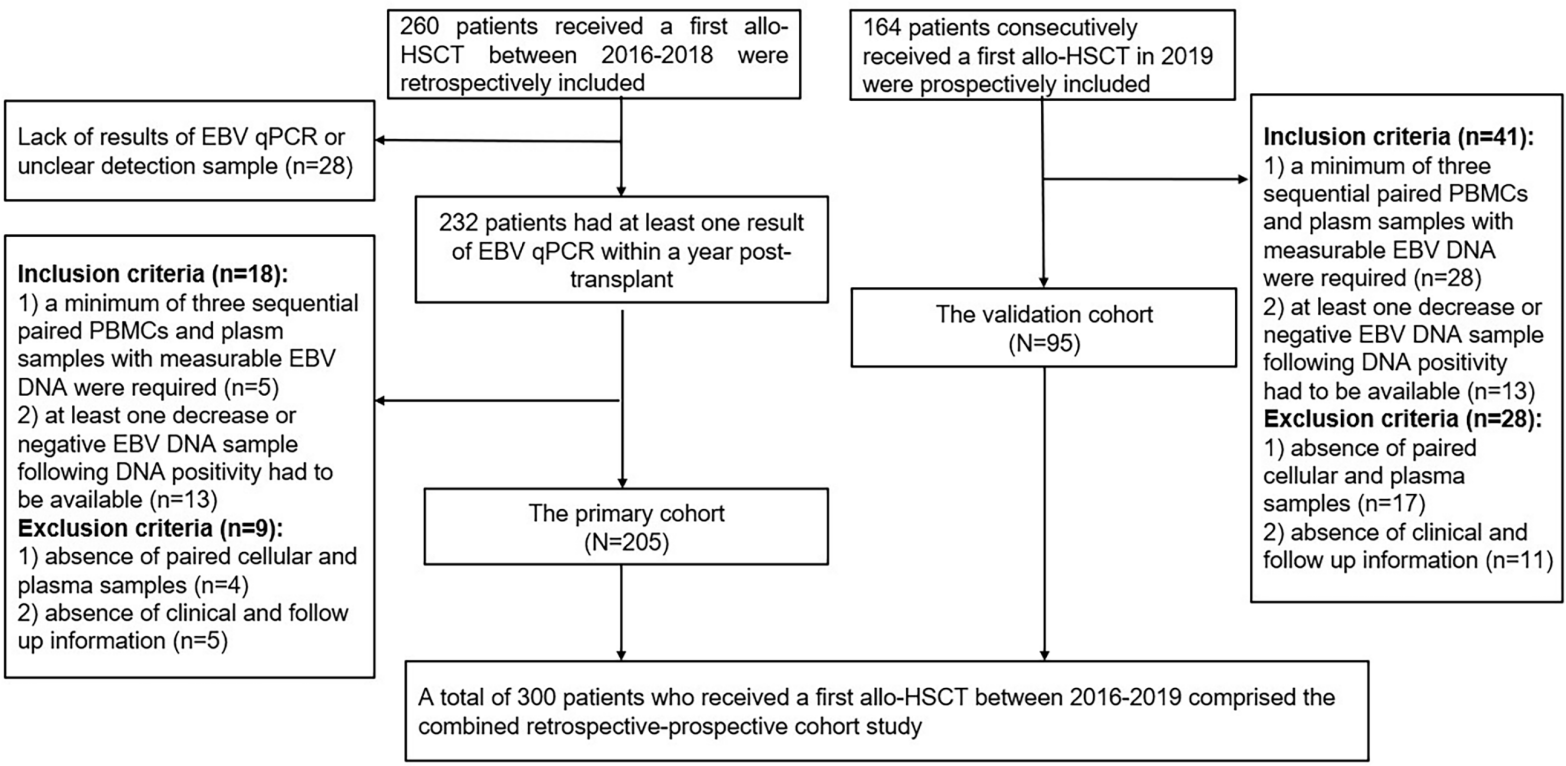
Flow diagram of patient specimens included in the study.

Patients received a first allo-HSCT at Wuhan Union Hospital from January 2016 to December 2018 were retrospectively collected as the primary cohort. Inclusion criteria were: 1) a minimum of three sequential paired PBMCs and plasma samples with measurable EBV DNA during the first-year post-transplant were required; 2) at least one decrease or negative EBV DNA sample following DNA positivity had to be available. The exclusion criteria were as follows: 1) absence of paired PBMCs and plasma samples; 2) absence of clinical and follow up information. Data of consecutive patients performed allo-HSCT in 2019 were prospectively collected as the validation cohort according to the above criteria. Flow diagram of included patients was shown in **Figure 1**. Medical record data included patient demographics, disease type, conditioning regimen, donor source, GVHD prophylaxis, CMV DNA, EBV DNA, time to peak EBV DNA load, lymphocyte subsets and cytokines results simultaneously detected at EBV DNA monitoring time points within the first year after HSCT.

### Transplantation procedure

Transplantation donor-recipient pairs were typed at the HLA-A, -B, -C, -DRB1, and -DQB1 using high-resolution technique. Donors were primed with recombinant human granulocyte colony-stimulating factor (rhG-CSF) at a dose of 8 to 10 ug/kg per day injected subcutaneously to mobilize BM and/or peripheral blood. Unmanipulated rhGCSF-primed peripheral blood stem cells and/or BM were infused into the recipient on the day of collection, with the target count for CD34+cells set at above 2×10^6^/kg of the recipient weight.

The majority of patients received myeloablative conditioning, including the modified Bu/Cy regimen and the modified TBI/Cy regimen. Patients who were intolerant to intensive chemotherapy received a reduced intensity conditioning regimen based on fludarabine, low-dose busulfan, cytarabine or cyclophosphamide according to the primary disease.

Graft-versus-host disease prophylaxis included the use of cyclosporin A (CsA) and short-term methotrexate for HLA-identical transplantation. A combination of CsA or tacrolimus, short-term MTX, mycophenolate mofetil and basiliximab was provided to haplo-HSCT recipients as GvHD prophylaxis. ATG (thymoglobulin, rabbit) was added for patients of haplo-HSCT. CsA 2.5 mg/kg per day was given as a continuous IV infusion from day 1 until patients were able to tolerate oral medication. Individualized dosage adjustment of CsA was based on plasma concentration to maintain a target dose of 150–250 ng/mL. CsA was progressively tapered by 5% weekly and discontinued after around 3–4 months in cases with no evidence of GvHD. MTX was administered intravenously at dosages of 15 mg/m2 on day +1 and 10 mg/m2 on days +3, +6 and +11. Mycophenolate mofetil (7.5 mg/kg, orally twice daily) was administered from day − 9, which was tapered to half until day +60 and was discontinued thereafter based on the presence or absence of severe GvHD, infectious diseases and relapse risk. Basiliximab was given intravenously at a dose of 20 mg by 30-minute IV infusion on day 0 (2 h before graft infusion) and day +4.

### EBV viral load monitoring and antiviral strategy

Patients underwent surveillance monitoring for EBV in peripheral blood within one week before transplantation and at least weekly up to 100 days post-transplantation and longer if they remained on immunosuppressive therapy or had detectable EBV DNA. A quantitative real-time PCR was performed using an EBV PCR quantitative diagnostic kit (Shengxiang Biotechnology, Hunan, China) and a Stratagene Mx3000P analyzer (Agilent Technologies, Germany). An EBV DNA level of > 400 copies/mL plasma or per 10^6^ PBMCs was defined as positive. EBV DNAemia was defined as the detection of EBV DNA (at any level) in one or more plasma specimens. The main criteria for clinical suspicion of PTLD were a significant increase in EBV DNA load with clinical symptoms including fever, lymphadenopathy, hepatosplenomegaly, tonsillitis, adenopathy, organomegaly or other end-organ manifestations. Tissue biopsy was required to diagnose proven EBV-PTLD (Styczynski et al., 2016).

Preemptive ganciclovir at a dose of 10 mg/kg/day was administered from −9 to −2 days to prevent virus infection. EBV DNAemia recipients at post-transplantation were treated individually or in combination with the following therapies including tapering of immunosuppressive agents, ganciclovir, foscarnet sodium and intravenous immunoglobulin. The treatment against EBV-PTLD included administration of rituximab, donor lymphocyte infusion, and EBV-specific cytotoxic lymphocyte infusion.

### Multiparametric flow cytometric analysis

Immune analysis was performed as described previously (Cai et al., 2021). Peripheral venous blood (2-4 mL) was collected in K3-EDTA anticoagulant tubes, then 100 uL of whole blood were incubated with fluorochrome-conjugated antibodies for 15 minutes at room temperature followed by incubation with 2 mL of BD lysing buffer at room temperature for 10 minutes to lyse erythrocytes. After washing once, the sample was resuspended in 300 mL of phosphate-buffered saline, and stored at 4°C until acquirement. Sample acquirement was performed on a BD FACSCanto flow cytometer, and data were analyzed using the Diva software. In addition, the whole blood was centrifuged at 300 g for 15 minutes and the serum was extracted and stored at 20°C before test. Multiple serum cytokines (IL-2, IL-4, IL-6, IL-10, TNF-a, and IFN-γ) were quantified using the Human Cytokine Kit (Jiangxi Saiji Bio-Tech, Jiangxi, China) following the manufacturer manual.

### Definitions

Neutrophil engraftment was defined as the first time that the absolute neutrophil count exceeded 0.5×10^9^/L for three consecutive days, platelet engraftment was defined as the first time the platelet count of >20×10^9^/L without transfusion support for seven consecutive days. Transplant-related mortality (TRM) was defined as death caused by all causes not related to the underlying disease. Overall survival (OS) was defined as the days from HSCT to death from any cause. Diagnosis and clinical grading of aGVHD and cGVHD were established according to the standard criteria (Przepiorka et al., 1995; Jagasia et al., 2015).

### Statistical analyses

Categorical variables were expressed as absolute and relative frequencies, and significance was detected using the Chi-squared or Fisher’s exact test. The quantitative variables were expressed as mean standard deviation, and significance was evaluated using Student’s t-test. Non-normally distributed variables were expressed in median and interquartile intervals (IQR), and significance was determined using the Kruskal-Wallis test. The receiver operator characteristic (ROC) curves were performed using MedCale Software to investigate predictive values, cutoff points were identified following Youden’s index. OS, Disease-free survival (DFS) and GvHD and relapse-free survival (GRFS) were estimated by using the Kaplan–Meier method and the curves of various subgroups were compared by the log-rank tests. The cumulative incidence (CI) method was used to estimate TRM and relapse, accounting for the presence of competing risks (Fine and Gray, 1999). Multivariate analysis was performed using Cox regression model, and all risk factors whose *p* values were below 0.1 in univariate analyses were included in multivariate. *p*<0.05 was considered statistically significant. SPSS 25.0 statistical software and R 3.6.3 package were used for statistical analysis.

## Results

### General patient characteristics and measurement for EBV DNA

Overall, 205 patients of the primary group and 95 patients of the validation group were enrolled in the present study, which included 241 malignant patients and 59 SAA patients. Most of patients received myeloablative conditioning. 210 (70%) patients received a graft from partially mismatched related donor, 80 (26.7%) received a graft from matched related donor and 10 (3.3%) received HSCT from matched unrelated donor. At the first year after transplantation, plasma EBV DNA positivity was observed in 44 (14.7%) patients, the median onset to the first detectable EBV DNA in plasma was 47 (39–79) days. PBMCs EBV DNA was detected in 272 patients (90.7%), and the median time at the peak load of PBMCs EBV DNA was 62 (IQR, 43-150) days. Transplant characteristics and EBV DNA test results of the primary and validation cohorts are summarized in **Table 1**.

**Table 1:** Clinical characteristics and EBV DNA results in the primary and validation cohort.

	Overall cohort (n = 300)	primary cohort (n = 205)	Validation cohort (n = 95)	*P* value
**Age, median (IQR)**	28 (6-62)	28 (6-62)	30 (9-57)	0.727
** Period**				0.988
Adult	237 (79)	162 (79)	75 (78.9)	
Children	63 (21)	43 (21)	20 (21.1)	
**Gender, n (%)**				0.747
Male	184 (61.3)	127 (62)	57 (60)	
Female	116 (38.7)	78 (38)	38 (40)	
**Disease, n (%)**				0.599
malignant	241(80.3)	163 (79.5)	78 (82.1)	
SAA	59 (19.7)	42 (20.5)	17 (17.9)	
**Conditioning regimen**				0.892
MAC	210 (70)	143 (69.8)	67 (70.5)	
RIC	90 (30)	62 (30.2)	28 (29.5)	
**Stem cell, n (%)**				0.591
PBSC	186 (62)	125 (61)	61 (64.2)	
PBSC+BM	114 (38)	80 (39)	34 (35.8)	
**Donor type, n (%)**				0.847
PMRD	210 (70)	144 (70.2)	66 (69.5)	
MRD	80 (26.7)	55 (26.8)	25 (26.3)	
MUD	10 (3.3)	6 (2.9)	4 (4.2)	
**ABO match, n (%)**				0.755
match	165 (55)	114 (55.6)	51 (53.7)	
mismatch	135 (45)	91 (44.4)	44 (46.3)	
**Co-infection with CMV, n (%)**	77 (25.7)	51 (24.9)	26 (27.4)	0.646
EBV DNA test results*				
Plasma negative, PBMCs negative, n (%)	30 (10)	21 (10.2)	9 (9.5)	0.836
Plasma positive, PBMCs negative, n (%)	0 (0)	0 (0)	0 (0)	—
Plasma negative, PBMCs positive, n (%)	226 (75.3)	157 (76.6)	69 (72.6)	0.460
Plasma positive, PBMCs positive, n (%)	44 (14.7)	27 (13.2)	17 (17.9)	0.282
Time to peak EBV loads in PBMCs, median (IQR), days	62 (143-150)	65 (44-168)	56 (42-100)	0.099
Time of plasma EBV DNA positive, median (IQR), days	47 (39-79)	44 (32-101)	50 (40-67)	0.426
Peak EBV loads in PBMCs^#^, median (range), copies/10^6^ cells	2.7×10^4^ (5.1×10^2^-4.3×10^7^)	2.6×10^4^ (5.1×10^2^-4.3×10^7^)	3.0×10^4^ (6.6×10^2^-1.2×10^7^)	0.540
EBV-PTLD, n (%)	10 (3.3)	7 (3.4)	3 (3.2)	0.908

SAA, severe aplastic anemia; RIC, reduced-intensity conditioning; EBV, Epstein-Barr virus; BM, bone marrow; PBSC, peripheral blood stem cells; PMRD, partially mismatched related donor; MRD, matched related donor; MUD, matched unrelated donor; MAC, myeloablative conditioning; CMV, cytomegalovirus; PTLD, post-transplant lymphoproliferative disorders; IQR, interquartile range.

*An EBV DNA level of > 400 copies/mL plasma or per 10^6^ PBMCs was defined as positive.

The analysis population was among the patients who tested EBV DNA positive in PBMCs.

### Probability of PTLD following allogeneic HSCT

**Figure 2:**
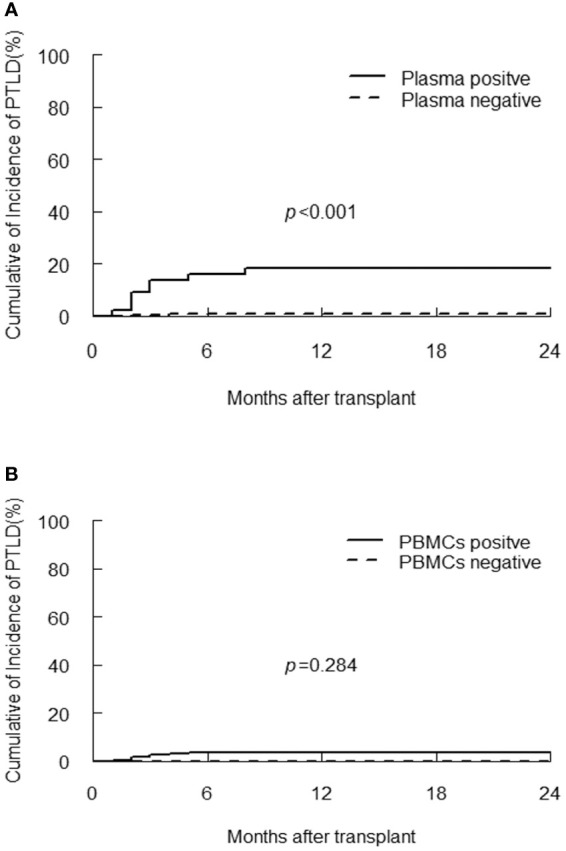
Cumulative incidence of PTLD according to EBV DNA detection in plasma **(A)** and PBMCs **(B)**.

PTLD developed in 10/300 (3.3%) of participants at median 2.5 (range, 1 to 8) months post-transplantation, with a case fatality rate of 50%. Donors were match related in two patients and haploidentical related in eight patients (**Supplementary Table 1**). Eight patients with PTLD had detectable plasma EBV DNA, the one-year CI of PTLD in plasma positive group was significantly higher than plasma negative group (18.2% vs 0.8%, *p*<0.001, **Figure 2A**), the probability of PTLD did not differ according to value of EBV DNA in PBMCs (*p*=0.248, **Figure 2B**). However, quantitative analysis of EBV DNA load in PBMCs demonstrated that the median peak viral load in the PTLD group was significantly higher than the non-PTLD group [1.1×10^6^ (range, 2.5×10^4^-4.3×10^7^) vs 1.8×10^4^ (range, 0-1.2×10^7^) copies/10^6^ cells, *p*<0.001].

### Diagnostic value of cut-off EBV DNA load in PBMCs for EBV-associated PTLD diseases in the primary and validation cohort

**Figure 3:**
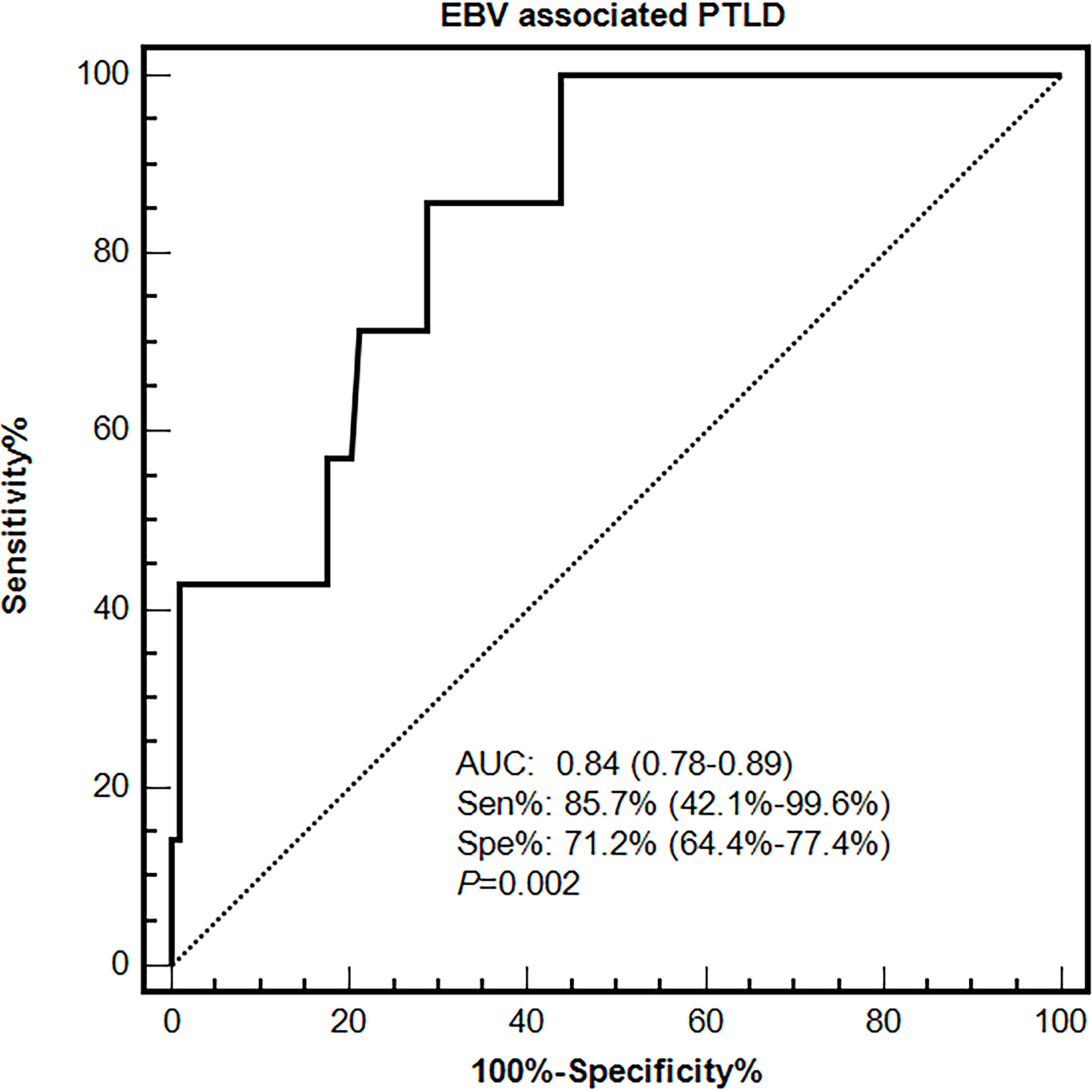
Diagnostic value of EBV DNA loads in PBMCs for EBV-associated PTLD disease in the primary cohort.

Primary and validation cohorts were applied to explore an effective cut-off value of EBV DNA load in PBMCs for the diagnosis of EBV-PTLD. In the primary cohort, the results of ROC curve analysis identified peak EBV DNA load in PBMCs as likely prognostic predictors with high AUC values [0.84 (95% CI 0.78-0.89)], associated with the optimal cut-off value was 7.3×10^4^ copies/10^6^ cells, the sensitivity and specificity were 85.7% (95% CI 42.1%-99.6%) and 71.2% (95% CI 64.4%-77.4%), respectively (**Figure 3**).

The performances of the cut-off value of PBMCs EBV DNA in the validation cohort were similar to that in the primary cohort, the sensitivity and specificity were 100% (95% CI 15.8%-100%) and 76.1% (95% CI 66.1%-84.4%), respectively. ROC curve analysis using the combination of the optimal cut-off value (7.3×10^4^ copies/mL) in PBMCs and the qualitative EBV DNA value (400 copies/mL) in plasma demonstrated an increased AUC of 0.94 (95% CI 0.87-0.98) with 100% sensitivity and 86% specificity for development of EBV-PTLD. When furtherly adjusting for the clinical information including gender, age and ATG use, AUC was 0.95 (95% CI 0.88–0.98), suggesting the good and stable discrimination of the prediction model (**Table 2**).

**Table 2:** Diagnostic value of the validation cohort including 3 cases of PTLD diseases and 92 cases of non-PTLD diseases.

Threshold	AUC	Sensitivity (%)	Specificity (%)	*P* value
7.3×10^4^ copies/mL	0.88 (0.84~0.94)	100 (15.8~100)	76.1 (66.1~84.4)	<0.001
Combined model*	0.94 (0.87~0.97)	100 (15.8~100)	86.0 (77.3~92.3)	<0.001
Covariate adjusted model^#^	0.95 (0.88~0.98)	100 (29.2~100)	86.0 (77~92.3)	<0.001

Data are presented as percentage (Ninety-five percent confidence intervals).

*Combine the optimal cut-off of EBV DNA load (7.3×10^4^ copies/10^6^ cells) in PBMCs and qualitative EBV DNA detection (400 copies/mL) in plasma to differentiate EBV-associated PTLD from non-PTLD diseases.

Covariate adjusted model includes relevant clinical information (gender, age and ATG use).

### Risk factors for PTLD incidence

EBV DNA level was furtherly classified as low, intermediate and high-level based on the results in plasma and PBMCs. Low-level was defined as negative in both plasma (<400 copies/mL) and PBMCs (<400 copies/10^6^ cells), intermediate-level was defined as low EBV DNA load (< 7.3×10^4^ copies/10^6^ cells) in PBMCs or high load (≥7.3×10^4^ copies/10^6^ cells) in PBMCs but negative in plasma, and high-level was defined as high load in PBMCs and positive in plasma. By univariate and multivariate analysis, the presence of moderate to severe cGVHD (HR 10.43, 90% CI 1.73-63.03, *p*=0.011) and high-level of EBV DNA (HR 18.5, 90% CI 3.82-89.49, *p*<0.001) were the independent risk factors for EBV-PTLD.

### Impact of EBV DNA level on transplant outcomes

**Figure 4:**
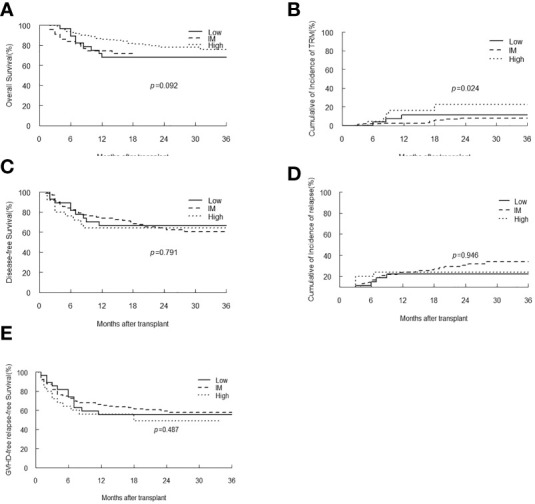
Overall survival (OS) **(A)**, transplant-related mortality (TRM) **(B)**, Disease-free survival (DFS) **(C)**, relapse **(D)** and GVHD-free, relapse-free survival **(E)** of malignant patients stratified by EBV-level.

The EBV-level variables were then subjected to the univariate and multivariate analysis of the transplant outcomes. Among 241 malignant patients, there were 27, 189, 25 patients who had low, intermediate and high-level of EBV DNA respectively. Median follow-up was 16.5 month (range 2-45). 58 (24.1%) patients died, relapse was the primary cause of death (46%) followed by GVHD (21.5%) and infection (16.9%) (**Table 3**). A total of 239 (99.2%) patients achieved neutrophil engraftment, and 234 (97.1%) had platelet engraftment. Neutrophil and platelet engraftment occurred in a median time of 11 days (range, 5–21) and 12 days (range, 6–39), respectively. Among the three EBV-level groups, there was no significant difference in the median time of neutrophil (*p*=0.225) and platelet (*p*=0.930) engraftment. The incidence of grades II to IV aGVHD on day +100 was 3.7%, 12.2% and 8% in low, intermediate and high EBV-level groups, respectively (*p*=0.399), the incidence of cGVHD was 14.8%, 9.5% and 12%, respectively (*p*=0.693). The estimated 3-year OS of malignant patients in high EBV-level group [60.2% (95% CI 42.3%-85.5%)] tended to be higher than low [66.7% (95% CI 51.1%-87%)] and intermediate [73.8% (95% CI 66%-82.4%)] EBV-level groups (*p*=0.097). The estimated TRM at 3 years of patients in high EBV-level group [22.7% (95% CI 20.9%-24.5%)] was significantly higher than low [11.1% (95% CI 10.4%-11.9%)] and intermediate [7.8% (95% CI 7.7%-8.0%)] EBV-level group (*p*=0.024). There was no significant statistical difference in DFS, relapse and GRFS among the three groups based on EBV DNA level (**Figure 4**).

**Table 3:** Cause of death.

Cause of Death	Overall patients (n = 65)	Malignant patients (n = 58)	SAA patients (n = 7)
**Relapse/progression, no. (%)**	29 (44.6)	28 (48.3)	1 (14.3)
**Infection, no. (%)**	11 (16.9)	8 (13.8)	3 (42.9)
**Graft failure, no. (%)**	1 (1.5)	0 (0)	1 (14.3)
**GVHD, no. (%)**	14 (21.5)	14 (24.1)	0 (0)
**TMA, no. (%)**	2 (3.1)	2 (3.4)	0 (0)
**PTLD, no. (%)**	5 (7.7)	3 (5.2)	2 (28.6)
**Hemorrhagic event, no. (%)**	3 (4.6)	3 (4.6)	0 (0)

GvHD, graft-versus-host disease; PTLD, post-transplant lymphoproliferative disorder.

**Figure 5:**
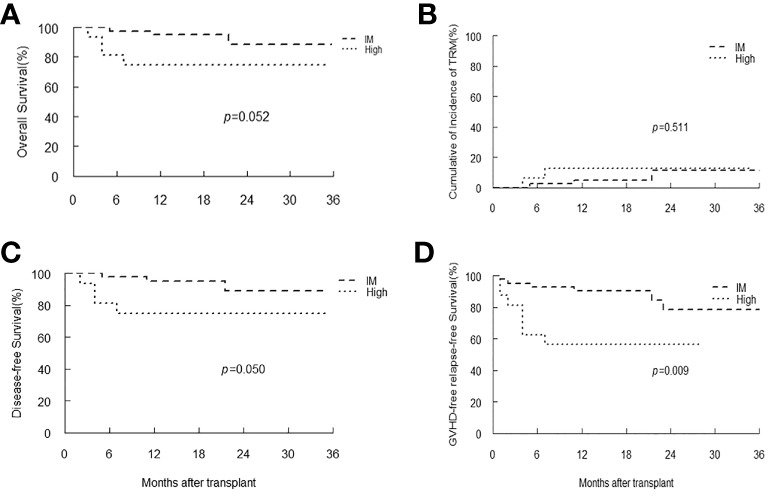
Overall survival (OS) **(A)**, transplant-related mortality (TRM) **(B)**, Disease-free survival (DFS) **(C)** and GVHD-free, relapse-free survival **(D)** of SAA patients stratified by EBV-level.

In patients with SAA, only one patient had low-level of EBV DNA load, and there were 42 and 16 patients classified as intermediate and high EBV-level group, respectively. With a median follow-up duration of 16 months (range 2-53), all patients achieved neutrophil engraftment, and 55 (93.2%) patients had platelet recovery. Seven patients died, and the most common cause of death was infection (42.9%). Kaplan-Meier analyses revealed that patients with intermediate-level of EBV DNA load tended to have longer OS, DFS and GRFS than that of patients with high-level (*p*=0.052, 0.05, 0.009, respectively). The TRM in intermediate EBV-level group was similar to that of high-level group (**Figure 5**).

In the entire cohort, EBV-level was not a prognostic factor for DFS, GRFS and relapse by univariate analysis. Multivariate analysis identified that poorer prognosis in terms of OS (HR 1.92, 95% CI 1.03-3.57, *p*=0.039) and TRM (HR 3.35, 95% CI 1.31-8.58, *p*=0.012) were observed in high-level of EBV DNA compared to intermediate-level (**Table 4**).

**Table 4:** Univariate and multivariate analyses for OS and TRM.

Variable	OS				TRM			
	Univariate analysis		Multivariate analysis		Univariate analysis		Multivariate analysis	
	HR (95% CI)	*p* value	HR (95% CI)	*p* value	HR (95% CI)	*p* value	HR (95% CI)	*p* value
**EBV DNA level**								
** Intermediate vs low**	0.53 (0.26-1.08)	0.082	0.53 (0.26-1.12)	0.097	0.47 (0.13-1.63)	0.232	0.56 (0.15-2.02)	0.373
** High vs low**	1.01 (0.43-2.37)	0.974	1.11 (0.46-2.71)	0.812	1.56 (0.40-6.05)	0.518	1.94 (0.47-7.97)	0.358
** High vs intermediate**	1.92 (1.03-3.57)	0.039	2.08 (1.10-3.96)	0.025	3.37 (1.34-8.44)	0.010	3.48 (1.35-8.97)	0.010
**Patient age**								
** ≥40 vs <40 yr**	0.90 (0.51-1.59)	0.727			0.13 (0.02-0.98)	0.115	5.09 (0.67-38.43)	0.115
**Patient gender**								
** Male vs female**	1.24 (0.74-2.05)	0.416			1.28 (0.54-3.02)	0.570		
**Disease type**								
** Malignant vs SAA**	2.07 (0.95-4,54)	0.069	1.99 (0.89-4.45)	0.096	0.90 (0.33-2.43)	0.837		
**Donor age**								
** ≥40 vs <40 yr**	1.04 (0.63-1.70)	0.886			1.26 (0.56-2.86)	0.576		
**Donor gender**								
** Male vs female**	1.36 (0.79-2.34)	0.266			2.41 (0.82-7.08)	0.110		
**D/P sex match**								
** F/M vs other**	0.89 (0.52-1.52)	0.663			0.94 (0.39-2.28)	0.886		
**ABO match**								
** Mismatch vs match**	1.08 (0.66-1.75)	0.771			0.82 (0.36-1.90)	0.645		
**HLA match**								
** Mismatch vs mismatch**	1.59 (0.88-2.87)	0.125			1.24 (0.49-3.14)	0.655		
**Conditioning**								
** MAC vs RIC**	0.85 (0.49-1.48)	0.574			1.15 (0.45-2.93)	0.766		
**Pretreatment with ATG**								
** Yes vs no**	1.18 (0.63-2.2)	0.613			1.29 (0.44-3.80)	0.642		
**Graft source**								
** PB vs PB+BM**	1.09 (0.66-1.91)	0.744			0.77 (0.34-1.76)	0.538		
**Platelet graft failure**								
** Yes vs no**	2.66 (1.07-6.64)	0.036	2.37 (0.91-6.18)	0.078	3.3 (0.77-14.15)	0.107		
**CMV infection**								
** Yes vs no**	1.74 (1.02-2.95)	0.039	1.35 (0.79-2.33)	0.275	2.07 (0.82-5.27)	0.125		
**Microbemia**								
** Yes vs no**	2.66 (1.07-6.64)	0.036	2.37 (0.91-6.18)	0.078	3.62 (1.34-9.75)	0.011	4.6 (1.62-13.07)	0.004
**Grade II-IV aGVHD**								
** Yes vs no**	2.31 (1.18-4.54)	0.015	2.39 (1.19-4.80)	0.014				
**Moderate-to-severe cGVHD**								
** Yes vs no**	2.07 (1.12-3.88)	0.023	1.75 (0.90-3.39)	0.098	4.77 (1.77-12.87)	0.002	5.97 (2.03-17.55)	0.001

SAA, severe aplastic anemia; RIC, reduced-intensity conditioning; EBV, Epstein-Barr virus; BM, bone marrow; PB, peripheral blood stem cells; MAC, myeloablative conditioning; CMV, cytomegalovirus; GvHD, graft-versus-host disease.

Additionally, we compared the survival outcomes and GVHD occurrence in patients with early peak EBV load in PBMCs within 3 months after transplantation to the patients whose EBV DNA load rose to peak during 3-12 months after transplantation. We observed that patients with a peak EBV DNA in PBMCs during 3-12 months after HSCT had a higher incidence of cGVHD at 1 year than those within 3 months after HSCT (17.4% vs 13.7%, *p*=0.029).

### Immunological features in patients with different level of EBV DNA load

Among the 300 patients enrolled in the study, eighty patients had lymphocyte subsets detection after HSCT, sixty of whom had cytokines detection concurrently. There was no significant difference in baseline characteristics between those who underwent immune analysis and those who did not (**Supplementary Table 2**). Moreover, the median time of immunodetection in the low, intermediate and high EBV-level groups was 60, 59 and 75 days, respectively (*p*=0.075). No significant difference was observed in the donor type among the three EBV-level group (*p*=0.537). The overall analysis indicated statistically lower CD4+ T cell percentage and CD4+/CD8+ ratio among the high EBV-level group in comparison with the low and intermediate EBV-level groups (*p*=0.002 and 0.001, respectively). The high EBV-level group showed abnormally increased CD8+ T cell percentage compared to the low and medium EBV-level groups (*p*=0.008), no significant difference found regarding the percentages of CD3+ cells, NK cells and B cells among the three EBV-level groups (**Table 5**). Cytokine profile analysis showed the level of IL-10 was significant higher in both high and low EBV-level groups than in the medium group (*p*=0.032), as is shown in **Table 6**.

**Table 5:** Frequency of immune cells subsets analysis.

Percentages of^#^ Immune cell subsets	Normal*range	Median (IQR) Low group(n =3 4)	Medium group (n = 38)	High group(n = 8)	*p* value
CD3+T cell (%)	58.17~84.22	80.1 (65.6~86.6).	75.2 (65.1~84.4)	85.7 (78.9~89.7)	0.172
CD4+T cell (%)	25.34~51.37	23.2 (13.4~30.0)	19.8 (11.1~26.4)	5.7 (9.4~11.4)	**0.002**
CD8+T cell (%)	14.23~38.95	46.2 (28.4~57.5)	46.7 (36.6~56.4)	71.4 (52.5~77.4)	**0.008**
B cell (%)	4.10~18.31	4.0 (1.4~12.2)	5.7 (2.0~10.5)	7.8 (4.7~12.7)	0.516
NK cell (%)	3.33~30.47	10.3 (5.8~19.9)	11.6 (3.9~18.3)	4.9 (3.5~8.3)	0.119
CD4+/CD8+ ratio	0.41~2.72	0.5 (0.2~0.9)	0.4 (0.3~0.6)	0.1 (0.08~0.2)	**0.001**

^#^The percentage of immune cells within total lymphocyte population were calculated. *Data are shown as the normal ranges of the indicators recommended by the reagent manual. Significant p values are represented in bold.

**Table 6:** Cytokine profiles analysis.

cytokines	Normal*Range (pg/ml)	Median (IQR) Low group (n = 22)	Medium group (n = 33)	High group (n = 6)	*p* value
IL2	≤5.71	3.6 (3.2~4.1)	3.3 (2.8~3.7)	3.4 (3.1~4.2)	0.112
IL4	≤3	2.8 (2.5~3.3)	2.8 (2.2~3.1)	2.5 (2.2~4.7)	0.682
IL6	≤5.3	10.3 (7.2~17.4)	10.2 (5.2~26.0)	11.5 (6.9~30.0)	0.850
IL10	≤4.91	7.3 (5.6~9.7)	5.2 (4.3~8.4)	9.0 (7.9~12.5)	**0.032**
TNF-α	≤4.6	3.1 (2.7~3.8)	2.9 (2.6~3.8)	3.0 (2.6~5.3)	0.704
INF-γ	≤7.42	3.6 (2.8~5.8)	3.0 (2.2~4.2)	3.7 (2.7~6.5)	0.242

*Data are shown as the normal ranges of the indicators recommended by the reagent manual. IOR, interquartile range. Significant p values are represented in bold.

## Discussion

To our knowledge, whether EBV DNA load in PBMCs has practical implications for assessing the risk of transplant outcome and the PTLD occurrence remains to be clarified. Based on the established primary and validation cohorts of the present study, the diagnostic cut-off value of EBV DNA load in PBMCs for EBV-associated PTLD was determined to be 7.3×10^4^ copies/10^6^ cells, and the combination of the cut-off value of EBV DNA in PBMCs and qualitative detection in plasma was expected to identify the EBV-PTLD better. Additionally, the results reminded us that once the high load of intracellular EBV DNA occurs, the frequency of EBV reactivation monitoring should be strengthened, and antiviral therapy should be considered after weighing the advantages and disadvantages, especially in the presence of coexisting plasma EBV DNA positivity. We expect prompt and effective prevention of impending EBV-PTLD after HSCT by using the EBV DNA threshold value.

Epstein-Barr virus (EBV) infection were significantly associated with poorer survival and higher TRM for HSCT patients (Styczynski et al., 2016; Ottaviano et al., 2020). Our findings demonstrated that patients with intermediate-level of EBV DNA load presented in PBMCs and plasma were associated with better prognosis in terms of OS and TRM after HSCT, especially when compared with the high EBV-level group. Similarly, Li et al. study suggested patients with intermediate level of EBV DNA load after HSCT might reflect balanced reconstitution of host immune response and had better OS than those with high (>90000 copies/10^6^ cells) or very low level (<6000 copies/10^6^ cells) of cellular EBV DNA (5-year OS 90% vs 67%, *p*<0.03) (Li et al., 2016). T cell dysfunction plays an important role in the reactivation and replication of EBV (Chen et al., 2020). It is likely that the presence of intermediate-level of EBV DNA load presented in PBMCs and plasma is a consequence of appropriate immunosuppression, in this case, effective clearance of T cells and better engraftment can be achieved, but it could also impair the control of viral load in latent EBV-infected cells, leading to the increased EBV DNA load in PBMCs. Undetectable or high level of latent EBV infection during the immune reconstitution early after transplantation may due to the inadequate or excessive immunosuppressive doses and reflect the exposure of an unbalanced control or homeostasis between the virus infection and the host immune system (Yu et al., 2021).

In the present study, we found that patients who showed an increase in PBMCs EBV DNA load and reached a peak level during 3-12 months after transplantation should be alert for the occurrence of cGVHD. Possible explanation for this phenomenon is that B-cell recovery occurs relatively late after HSCT (usually after day 100), and then it can be activated by EBV infection which is considered to play a role in cGVHD pathogenesis (Allen et al., 2012). Accordingly, when patients develop a sharp rise in PBMCs EBV DNA load after allo-HSCT, reduction or discontinuation of immunosuppressor may result in poor prophylaxis and control of GVHD (Ru et al., 2020), while the anti–B cell immunotherapy such as rituximab may effectively reduce the viral load as well as the occurrence of cGVHD (Zhu et al., 2019).

EBV can induce cellular immune responses and influence the immune recovery in the infected immunocompromised host (Rickinson et al., 2014). Immunoassays of the present study showed a poor immune reconstitution of high EBV-level group as reflected in profoundly lower proportion of CD4+ T cells and abnormally elevated CD8+ T cell percentage within one-year after HSCT. In line with the present study, Chen et al. concluded that the EBV DNA copy number was positively correlated with the level of CD8+ T cells and IL-10, the percentage of CD4+ T cells and the ratio of CD4+/CD8+ cells were lower in the EBV-positive group than in the EBV-negative group (Chen et al., 2020). Moreover, low numbers of endogenous CD4+ T cells will in turn increase the risk of the development of EBV-associated diseases in immunosuppressed patients (Mautner and Bornkamm, 2012). Also, our results revealed that IL-10 were present at significantly higher level in both low and high EBV-level groups than the reference value, whereas the level of IL-10 in the medium EBV-level groups was within the reference value range. As a kind of immunosuppressive cytokine, IL-10 participates in suppressing the activity of antivirus immune cells involving NK cells and macrophages (Lee et al., 2011), and it has been widely reported to be expressed in EBV-positive lymphomas, which hinder virus clearance and facilitate EBV proliferation. Accumulating evidence confirms that T-cell depletion strategies including ATG attribute the high rates of virus reactivation prominently (Liu et al., 2018). Hence, Dynamic monitoring of cytokines and the reconstitution of T lymphocytes in conjunction with EBV DNA load may be useful as biomarkers in improving the prediction of PTLD development and prognosis (Althubaiti et al., 2019).

There are still several limitations in our study. This was a small single-center study that included the very small numbers of PTLD, and we did not monitor virus-specific lymphocyte reconstitution which could be important in evaluating the immune situation of those patients with EBV reactivation better. Despite several limitations, the present study emphasized the role of EBV DNA in both PBMCs and plasma as predictors of suspected EBV-PTLD and significant impact on transplant prognosis. These data, when confirmed by a larger prospective study, may not only facilitate personalized management of EBV infection in allo-HSCT recipients but also provide monitoring strategy to other types of patients with EBV-related disease.

## Data availability statement

The raw data supporting the conclusions of this article will be made available by the authors, without undue reservation.

## Ethics statement

The studies involving human participants were reviewed and approved by ethics committee of Union Hospital, Tongji Medical College, Huazhong University of Science and Technology. The informed consent for collecting general information and routine laboratory test results of all patients was waived by the Ethics Committee.

## Author contributions

HW, YH and YY: study conception and design, obtaining funding, providing feedback on the report; ZX, QL, PY, ZZ, WS and HY: acquisition and analysis of the data, updating reference lists; XZ, XL and JH: interpretation of data and drafting of the work; all authors: final approval and agreement to be accountable for all aspects of the work in ensuring that questions related to the accuracy or integrity of any part of the work are appropriately investigated and resolved.

## Funding

This project was supported by the National Natural Science Foundation of China (grant no. 81770134).

## Acknowledgments

We extend our gratitude to the contributions of the researchers and all the patients who participated in the present study.

## Conflict of interest

The authors declare that the research was conducted in the absence of any commercial or financial relationships that could be construed as a potential conflict of interest.

## Publisher’s note

All claims expressed in this article are solely those of the authors and do not necessarily represent those of their affiliated organizations, or those of the publisher, the editors and the reviewers. Any product that may be evaluated in this article, or claim that may be made by its manufacturer, is not guaranteed or endorsed by the publisher.
