# Supplementary material for: Clinical value of plasma and peripheral blood mononuclear cells Epstein–Barr Virus DNA dynamics on prognosis of allogeneic stem cell transplantation
Source: Front Cell Infect Microbiol. 2022 Sep 16;12:980113. doi: 10.3389/fcimb.2022.980113 (PMC9524571; doi:10.3389/fcimb.2022.980113)
Supplement: Supplementary file 2 [file Table_2.docx]

**Supplementary Table 2.** Baseline characteristics of the included and excluded patients of the total cohort in immune cells subsets and cytokines profiles analysis.

|  | **Included cohort (n=80)** | **Excluded cohort (n=220)** | ***P* value** |
| --- | --- | --- | --- |
| **Age, median (range)** | 28 (7-54) | 29 (6-62) | 0.727 |
| **Sex, n (%)** |  |  | 0.292 |
| Male | 53 (66.3) | 131 (59.5) |  |
| Female | 27 (33.8) | 89 (40.5) |  |
| **Conditioning regimen** |  |  | 0.569 |
| MAC | 54 (67.5) | 156 (70.9) |  |
| RIC | 26 (32.5) | 64 (29.1) |  |
| **ATG use** |  |  |  |
| yes | 68 (85) | 172 (78.2) | 0.192 |
| no | 12 (15) | 12 (15) |  |
| **Stem cell, n (%)** |  |  | 0.076 |
| PBSC | 43 (53.8) | 143 (65) |  |
| PBSC+BM | 37 (46.3) | 77 (35) |  |
| **HLA matched loci, n (%)** |  |  | 0.067 |
| 5/10 | 42 (52.5) | 84 (38.2) |  |
| 6-8/10 | 26 (32.5) | 66 (30) |  |
| 9-10/10 | 17 (21.3) | 70 (31.8) |  |
| **ABO match, n (%)** |  |  | 0.793 |
| match | 45 (56.3) | 120 (54,5) |  |
| mismatch | 35 (43.8) | 100 (45.5) |  |
| **Co-infection with CMV, n (%)** | 48 (60) | 120 (54.5) | 0.400 |

Abbreviations: MAC, myeloablative conditioning; RIC, reduced-intensity conditioning;

R, recipient; BM, bone marrow; PBSC, peripheral blood stem cells; CMV, cytomegalovirus;
